# Social Processes in Late-Life Suicide

**DOI:** 10.1093/geroni/igaa057.2114

**Published:** 2020-12-16

**Authors:** Elizabeth Necka

## Abstract

Suicide is the tenth leading cause of death in the United States, and social isolation – either real or perceived – is one of the primary risk factors for a suicidal attempt (Calati et al.,2019). Late adulthood is characterized by both rapid increases in both social isolation (Cornwell,2011) and loneliness (i.e., perceived social isolation; Hawkley, Wroblewski, Kaiser, Luhmann, & Schumm,2019), which enhance risk of mental disorders (Santini et al.,2020), as well as by suicide rates that are higher than in any other age group (SAMSHA,2017). What are the mechanisms by which social isolation confers risk (and social connection confers resilience) to suicidal thoughts and behaviors in aging, and what promising interventions exist for addressing social impediments in older adulthood? What barriers exist to providing services to socially isolated older adults contemplating suicide, and what are the public health implications of social isolation and suicide in late life? This symposium will feature talks on the role of social motivation and empathy in the development of (or resilience to) suicidal ideation in older adults, on interventions that draw upon the Interpersonal Theory of Suicide and utilize social engagement and digital ‘mHealth’ services to reduce late-life social isolation, depression, and suicidal ideation, and on National Institute of Mental Health funding priorities and efforts to address suicide. After attending this session, participants will be able to articulate mechanisms by which social isolation confers risk for suicide in older adulthood and to identify opportunities and obstacles for effective intervention implementation.

